# Near telomere-to-telomere genome assembly enables the discovery of introgressed purple-leaf gene *BraMYB114* in pak choi (*Brassica rapa* subsp. *chinensis*)

**DOI:** 10.1093/hr/uhag193

**Published:** 2026-05-13

**Authors:** Daozong Chen, Xilin Gan, Yuanhui Song, Haidong Chen, Yi Liu, Shubei Wan, Bo Zhu, Zhanjun Lu, Qinyong Yang, Xinyi Guo, Liwei Gao, Martin A Lysak, Chen Tan

**Affiliations:** College of Life Sciences, Gannan Normal University, Ganzhou, China; College of Life Sciences, Gannan Normal University, Ganzhou, China; Ganzhou Sub-Center of National Vegetable Quality Standards Center, Ganzhou, China; College of Life Sciences, Gannan Normal University, Ganzhou, China; College of Life Sciences, Gannan Normal University, Ganzhou, China; College of Life Sciences, Gannan Normal University, Ganzhou, China; College of Life Sciences, Gannan Normal University, Ganzhou, China; College of Life Sciences, Gannan Normal University, Ganzhou, China; National Key Laboratory of Crop Genetic Improvement, National Research Center of Rapeseed Engineering and Technology, Huazhong Agricultural University, Wuhan, China; Central European Institute of Technology (CEITEC), Masaryk University, Brno, Czech Republic; College of Life Sciences, Gannan Normal University, Ganzhou, China; Central European Institute of Technology (CEITEC), Masaryk University, Brno, Czech Republic; Department of Experimental Biology, Faculty of Science, Masaryk University, Brno, Czech Republic; College of Life Sciences, Gannan Normal University, Ganzhou, China

## Abstract

Pak choi (*Brassica rapa* subsp. *chinensis*, AA genome) is a widely cultivated leafy vegetable that comes in green and purple varieties. The enrichment of anthocyanins enhances its nutritional value, making it highly preferred by consumers. However, the genes responsible for the purple phenotype in pak choi remain mostly unknown. Here, we assembled a near-complete telomere-to-telomere genome of the ‘ZBC’ variety using PacBio HiFi, HiC, and Illumina sequencing technologies. The assembled genome is 420.97 Mb and contains 51 387 genes. Through genomic variation and evolutionary analysis, we identified an introgressed fragment of 854.53 kb on chromosome A03, which originates from *B. carinata*. The BSA-seq and fine-mapping of the purple trait revealed that the target region overlaps with the 854.53-kb introgressed fragment by 390 kb. Functional annotation of the 390-kb interval revealed 48 genes, including *BraA03G007112*, an *MYB114*-like transcription factor named *BraMYB114*. It positively regulates anthocyanin biosynthesis by activating *BraDFR.A09* and *BraANS.A01* genes, as shown by transcriptomic, metabolomic, and quantitative real-time PCR analyses. Our research is the first to demonstrate that the *BrMYB114* gene, carried by an introgressed fragment, positively regulates anthocyanin synthesis in *B. rapa*. These findings advance our understanding of the *B. rapa* genome, introduce approaches for investigating key trait genes in introgression lines, and provide valuable targets for developing anthocyanin-rich vegetables.

## Introduction

The genus *Brassica* includes numerous important vegetables, such as Chinese cabbages (*B. rapa* subsp. *pekinensis*), pak choi (*B. rapa* subsp. *chinensis*), mustard greens (*B. juncea*), brussels sprouts (*B. oleracea* var. *gemmifera*), cabbages (*B. oleracea* var*. capitata*), collard greens (*B. oleracea* var. *acephala*), broccoli (*B. oleracea* var. *italica*), cauliflowers (*B. oleracea* var. *botrytis*), turnip (*B. rapa* subsp. *rapa*), rutabaga (*B. napus* var. *napobrassica*), and kohlrabi (*B. oleracea* var. *gongylodes*), as well as oilseeds like rapeseed (*B. napus*) and Indian mustard (*B. juncea*), and ornamental crops such as kale (*B. oleracea* var. *acephala*). This genus comprises six species, including three diploid species: *B. rapa* (AA, 2*n* = 20), *B. nigra* (BB, 2*n* = 16), and *B. oleracea* (CC, 2*n* = 18). Natural hybridization among these three species has resulted in the formation of three allotetraploid species: *B. juncea* (AABB, 2*n* = 36), *B. napus* (AACC, 2*n* = 38), and *B. carinata* (BBCC, 2*n* = 34). The genomic relationships among the three diploid and three allopolyploid *Brassica* species were described as U’s triangle [1].

Through prolonged natural selection and human domestication across various ecological zones, numerous subspecies of *B. rapa* have emerged, exhibiting significant phenotypic and genomic variation. These subspecies include Chinese cabbage (subsp. *pekinensis*), nonheading Chinese cabbage (subsp. *chinensis*), turnip (subsp. *rapa*), rapini (subsp. *sylvestris*), yellow sarson types (subsp. *trilocularis*), and the oilseed types (subsp. *oleifera*) [2–4]. Nonheading Chinese cabbage can be further classified into five varieties: pak choi (var. *communis*), Caitai (var. *tsai-tai*), Taicai (var. *tai-tsai*), Tacai (var. *rosularis*), and Fenniecai (var. *multiceps*) [5]. One notable variant is purple pak choi (‘ZBC’), which is distinguished by its deep purple leaves. This coloration is due to a high content of anthocyanins, compounds known for their potent antioxidant and anti-inflammatory properties. These benefits contribute positively to several aspects of health, including cardiovascular, neurological, and metabolic health [6, 7].

Chinese cabbage (‘Chiifu-401-42’) was the first *Brassica* species to have its genome sequenced [8], with several later updates [9–11]. This genome sequence has provided extensive information for the subsequent analysis of other *Brassica* species and serves as a reference genome for discovering genes associated with important traits in *B. rapa*. Subsequently, chromosome-level genomes have been reported for several varieties, including yellow sarson ‘Z1’ [12], nonheading Chinese cabbage ‘NHCC001’ [5], ‘ZYCX’ [13], ‘PC-fu’ [4], ‘XiangQingCai’ [14], and ‘wucai’ [15]. Other notable reports include Chinese cabbage varieties ‘A03’ [16] and ‘DH40’ [17], as well as turnip variety ‘ECD04’ [18]. Recently, a telomere-to-telomere (T2T) genome assembly was completed for Chinese cabbage ‘Chiifu-401-42’ [19] and ‘Futian’ [20]. Additionally, two nonheading Chinese cabbage varieties, AiJiaoBai (*B. rapa* subsp. *chinensis*, ‘AJ’) and HongShanCaiTai (*B. rapa* subsp. *purpuraria*, ‘HS’), have also had their genomes published [21]. The genomes of these *B. rapa* varieties have significantly facilitated the discovery of genes associated with specific agronomic traits, including clubroot resistance [17, 18], flowering time [13], and glucosinolates [16].

*B. rapa* includes a wide variety of purple anthocyanin-rich varieties. These anthocyanins not only give the leaves, stems, and midribs a striking reddish-purple hue but also enhance the nutritional value of these varieties due to their powerful antioxidant properties. Thus, identifying the genes that regulate anthocyanin biosynthesis in *B. rapa* is essential for the targeted breeding of high-quality anthocyanin-rich varieties. The first *anthocyaninless* (*anl*) purple trait was mapped to the R9 linkage group of rapid-cycling *B. rapa* [22]. Subsequently, the gene *BrPur*, which controls the purple leaves of *B. rapa*, was located on linkage group A03 [23]. Then, *BrTT8* [24], *BrMYB2* [25], *bHLH49* [26], *BrMYB73* [27], *BrMYBL2.1* [28], *BrPAP1a* [29], and *BrMYB2* [21, 30] were considered as key genes regulating the purple trait in *B. rapa*. Moreover, the genes *BraANS.A03* [31] and *BraCHI* [32], encoding catalytic enzymes in the anthocyanin biosynthesis pathway, were both identified on chromosome A03 in ‘ZBC’. However, there is currently no high-quality genome available for ‘ZBC’ to identify the genes regulating the purple leaf coloration, which would aid in breeding new varieties rich in anthocyanins.

Here, we report a near gap-free T2T genome of ‘ZBC’ generated using PacBio HiFi, HiC, and Illumina sequencing. When comparing the ‘ZBC’ genome with the previously published ‘HS’ and ‘AJ’ genomes, we identified an 854.53-kb introgressed fragment on chromosome A03. Further analysis revealed that this fragment originated from the allotetraploid *B. carinata* (2*n* = 34, BBCC). Using BSA-sequencing and map-based cloning, the region associated with the purple trait has been narrowed down to a 390-kb region. Gene annotation shows that this region contains 48 genes, among which only *BraA03G007112* (*BraMYB114*) has been identified as a *MYB114-like* transcription factor that may play a role in anthocyanin biosynthesis. The *BraMYB114* gene was identified as a key regulator of anthocyanin biosynthesis in ‘ZBC’, and the specific molecular markers were developed for breeding of purple *B. rapa* varieties. These findings clarify the molecular mechanisms by which *BraMYB114* regulates anthocyanin biosynthesis and offer valuable genomic resources for the improvement and breeding of *Brassica* crop varieties.

## Results

### Genome sequencing, assembly, and assessment of ‘ZBC’

We utilized 32.72 Gb Illumina sequencing data (~73× genome coverage) for *k-mer* analysis (*k* = 17), which allowed us to estimate the genome size of ‘ZBC’ to be approximately 456 Mb (Fig. S1). Comparing the results of our *k-mer* analysis with the published genome size of *B. rapa* (350.34–441.72 Mb, Table S1), we estimate that the genome size of ‘ZBC’ is nearly 450 Mb. Additionally, we sequenced 46.27 Gb of PacBio HiFi data (~103×), 83.98 Gb of Hi-C data (~187×), 11.51 Gb of Illumina RNA-seq data, and 13.23 Gb of PacBio Iso-seq (RNA-seq) data (Table S2).

First, we assembled the genome into contigs using PacBio HiFi sequencing data, achieving a contig N50 of 36.44 Mb (Table 1). These contigs were subsequently corrected and refined with Illumina reads. Further assembly was conducted using Hi-C data, which resulted in 10 pseudochromosomes and a total genome length of 420.97 Mb. The scaffold N50 reached 44.69 Mb, and the GC content was 37.41% (Fig. 1B; Table 1). This total genome length exceeds that of previously assembled genomes of two pak choi varieties, ‘PC-fu’ (411.40 Mb, [4]) and ‘ZYCX’ (370.42 Mb, [13]) (Table S2). A circos plot of the near T2T genome assembly was generated to visualize chromosome size, gene density, GC density, density of repetitive sequences, and interchromosomal collinearity (Fig. 1C). Chromosomes A02, A05, and A10 each contain one gap, A01 has two gaps, and A04 has three gaps that cannot be connected (Table 1, Table S3; Fig. S2).

**Table 1:** The assembly and annotation information of ‘ZBC’ genome.

**Assembly feature**	
No. of contigs	18
N50 of contig (Mb)	36.44 (longest:70.51)
Genome assembly (Mb)	420.97
GC content (%)	37.41
No. of pseudochromosomes	10
N50 of scaffold (Mb)	44.69
No. of gaps	8
No. of telomeres	20
No. of predicted centromeres	10
Repetitive region (%)	52.34
Gene number	51 387
Average gene CDS length (bp)	1121
Average gene length (bp)	2283
Average exon per gene	4.98
Functional annotation (%)	97.68
BUSCO (% of complete)	99.4
LTR Assembly Index (LAI)	25.84

**Figure 1:**
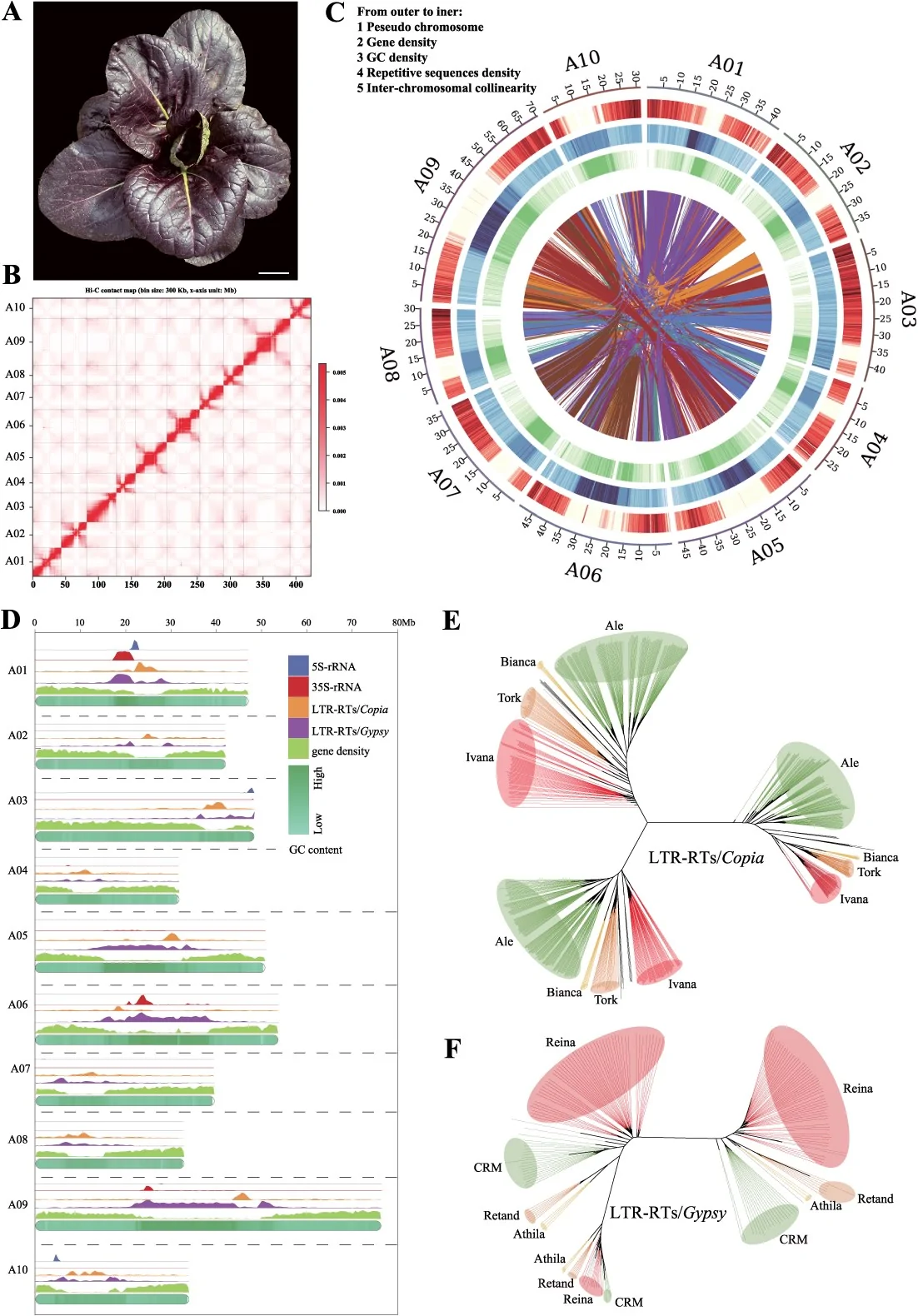
Genome assembly, chromosome sequence profiles, and transposable element abundances in the ‘ZBC’ genome. (A) The phenotype of the ‘ZBC’ cultivar of *B. rapa*. Scale bar, 5 cm. (B) Hi-C interaction heatmap for all 10 chromosomes. Bin size: 0.3 Mb, *x*-axis unit: Mb. (C) Circos plot of the ‘ZBC’ genome assembly. From the outer to the inner circle: chromosome size (Mb), gene density, GC density, repetitive sequences density, and interchromosomal collinearity (window size 0.5 Mb). (D) Chromosome-level distribution profiles of gene density, repeat density, and GC content (0.5-Mb sliding windows). (E) Phylogenetic analysis of *Copia* superfamily LTR-RTs. (F) Phylogenetic analysis of *Gypsy* superfamily LTR-RTs. Phylogenetic trees were constructed from all *Copia*/*Gypsy* superfamily LTR-RT sequences using the RAxML software with a maximum likelihood method.

The quarTeT toolkit was used to determine the telomeres. In total, 20 telomeres were identified across the 10 chromosomes. The telomere lengths ranged from 1.30 to 11.12 kb, with the shortest on the long arm of chromosome A05 and the longest telomere located on the short arm of chromosome A09 (Table S4; Fig. S2). Furthermore, centromeres were mapped using the Centro-Miner module of quarTeT. We successfully located the centromeres of all chromosomes, which ranged in size from 0.96 to 9.05 Mb. The longer centromeres (>5 Mb) were identified on chromosomes A10, A07, A02, A08, and A04, while chromosome A06 has the shortest centromere (0.96 Mb) (Table S5; Fig. S2). We also performed a similarity interaction analysis of the tandem repeat sequences in the centromere and near-centromere regions (Fig. S3).

The annotation of repetitive sequences in the ‘ZBC’ genome utilized both homology-based alignment and *de novo* prediction methods. The identified repetitive sequences constituted 52.34% (220.35 Mb) of the total genome, predominantly consisting of transposable elements (TEs) (Fig. S4). Long terminal repeat retrotransposons (LTR-RTs) were the most prevalent, representing 19.50% of the genome (Table S6). *Gypsy* superfamily LTR-RTs emerged as the most abundant TEs, followed by *Copia* superfamily LTR-RTs (Table S6). Notably, *Gypsy* LTR-RTs are predominantly located in the pericentromeric regions, whereas *Copia* LTR-RTs tend to be more concentrated in the centromeric regions (Fig. 1D; Fig. S5), which is consistent with the distribution of *Gypsy*- and *Copia*-type LTR-RTs in the centromeric and pericentromeric regions of *Brassica* species [20]. An evolutionary analysis of *Copia* and *Gypsy* LTR-RTs indicated that they can be broadly categorized into three clades (Fig. 1E and F). Among *Copia* LTR-RTs, Ale family exhibits the highest prevalence, maintaining a consistent distribution across all three clades (Fig. 1E). Conversely, among *Gypsy* LTR-RTs, Renia family is most prevalent (Fig. 1F).

By integrating *de novo* predictions, homology predictions from *Brassica* species (*B. juncea*, *B. napus*, and *B. rapa*), and transcriptome predictions, we identified a total of 51 387 protein-coding genes (Table S7). To assess the consistency of the genome, the Illumina reads were mapped to the genome assembly. The results showed an overall alignment rate of 99.40%, covering 99.30% of the annotated genes (Table S8). The high accuracy of the genome assembly was confirmed by Benchmarking Universal Single-Copy Orthologs (BUSCO) analysis, which indicated that 99.40% of genes were completely assembled (Table S8). Additionally, *k-mer* statistical analysis revealed a QV value of 72.64 for the genome, with chromosome-specific QV values ranging from 68.79 to 77.29 (Table S9). The LTR Assembly Index (LAI) for the ‘ZBC’ genome is 25.84, exceeding all published *B. rapa* genomes (Table 1).

Furthermore, we conducted a *de novo* prediction of noncoding RNAs and identified various types of ncRNA utilizing the Rfam database. In total, we identified 1378 snRNAs, 423 miRNAs, and 6 sRNAs, with snRNAs being the most abundant category. The average lengths of these RNAs were 103, 126, and 229 bp, respectively (Table S10). Collectively, these findings suggest that the assembled ‘ZBC’ genome is of high quality, reliable, and accurate.

### Comparative genomics analysis

To elucidate the phylogenetic relationships among ‘ZBC’ and other intraspecific taxa of *B. rapa* and other *Brassica* species, a comparative genomics analysis based on 1868 shared single-copy orthologous gene families was conducted (Fig. 2A). This analysis demonstrated that six *B. rapa* subspecies clustered within the same clade, diverging from the closely related species *B. oleracea* ~6.18 million years ago (Mya). These six *B. rapa* subspecies were further subdivided into three subclades, with the ‘ZBC’ being closely related to Chiifu, which diverged ~1.82 Mya (see also Fig. S6). An analysis of gene family evolution indicated that 539 gene families expanded (Table S12), while 163 gene families contracted in the ‘ZBC’ (Fig. 2A). A Kyoto Encyclopedia of Genes and Genomes (KEGG) enrichment analysis of the 539 expanded gene families revealed that they were enriched in pathways related to autophagy—other, plant hormone signal transduction, ascorbate and aldarate metabolism, amino sugar and nucleotide sugar metabolism, and the biosynthesis of various plant secondary metabolites (Fig. S7). To further investigate the evolutionary history of the ‘ZBC’, we estimated the distributions of intragenomic and interspecific homolog *Ks* (synonymous substitutions per site). The paralogs of ‘ZBC’ exhibited similar *Ks* values compared to other species in the Brassicodae (Fig. 2B). Among the six *B. rapa* varieties, the interspecific homologs were predominantly observed at a *Ks* value of 0.03, with an additional peak at 0.32 (Fig. 2C). This variation is attributed to the different timing of homologous gene fixation in the genome, resulting from the Brassiceae-specific genome triplication event [8]. In contrast, the interspecific homologs of ‘ZBC’ with *B. oleracea*, *B. nigra*, *R. sativus*, and *O. violaceus* were most abundant at *Ks* values of 0.06, 0.19, 0.20, and 0.27, respectively. This observation aligns with phylogenetic findings, showing that a more distant relationship with ‘ZBC’ corresponds to a higher *Ks* value (Fig. 2C).

**Figure 2:**
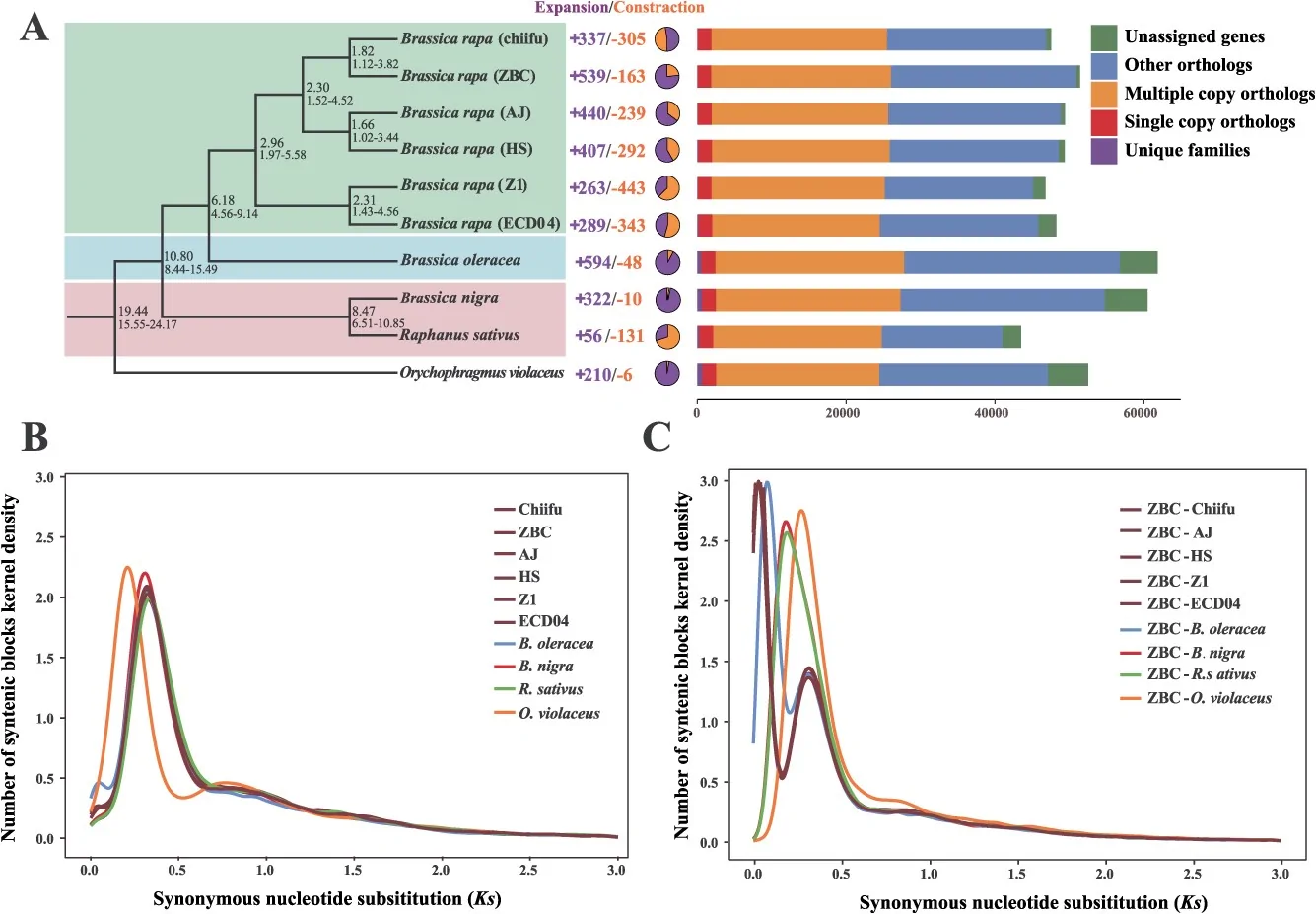
Evolutionary and comparative analysis of the ‘ZBC’ genome. (A) The phylogenetic tree and estimation of divergence time for ‘ZBC’ and other nine taxa. The middle panel displays the expansion/contraction of gene families in these taxa, and the right panel displays the number of gene classes. (B) The distribution of synonymous substitution rates (*Ks*) across collinear gene pairs. (C) The distribution of *Ks* values among collinear genes between ‘ZBC’ and the other nine taxa.

### Genome-wide identification of structural variation

To analyze the genomic sequence variants between ‘ZBC’ and other *B. rapa* subspecies, we compared the ‘ZBC’ genome with those of ‘AJ’ (*B. rapa* subsp. *chinensis*) and ‘HS’ (*B. rapa* subsp. *purpuraria*). These included single-nucleotide polymorphisms (SNPs), insertions and deletions (indels), and structural variants (SVs) (Table S13). The results showed that the ‘ZBC’ and ‘AJ’ genomes share a sum of 316.78 Mb of collinear regions, which are differentiated by 60 inversions (total length 16.49 Mb), 3280 translocations (11.63 Mb), and 245 duplications (7.79 Mb) (Fig. 3A; Table S13). The ‘ZBC’ and ‘HS’ genomes shared only 281.61 Mb of collinear regions, which contained 83 inversions (total length 22.24 Mb), 3951 translocations (14.12 Mb), and 495 duplications (2.90 Mb) (Fig. 3A; Table S13).

**Figure 3:**
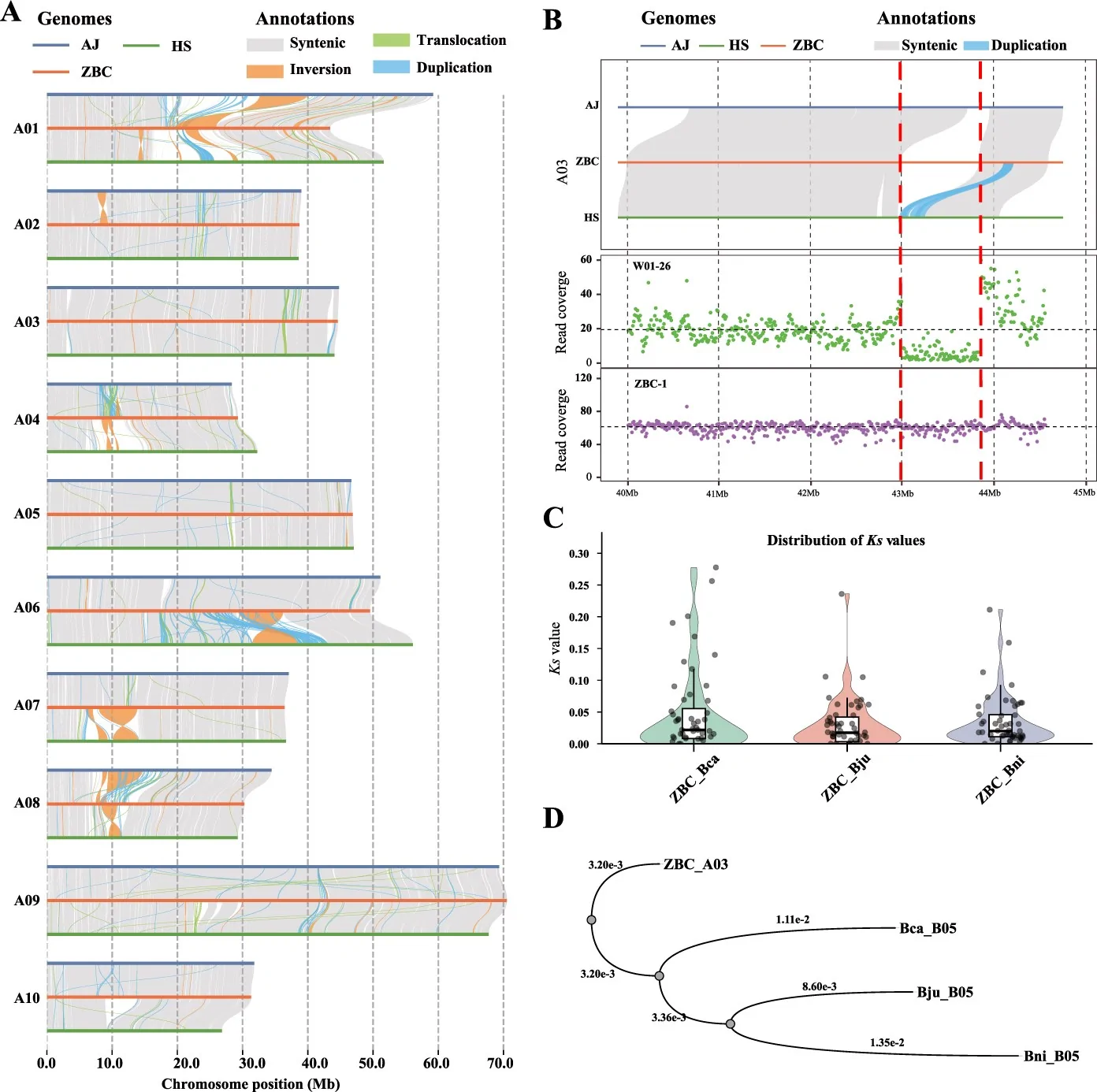
An 854.53-kb introgressed fragment has been identified on the short arm of chromosome A03 in ‘ZBC’. (A) Synteny and collinearity analysis among the ‘ZBC’, ‘AJ’, and ‘HS’ genomes. (B) Synteny and collinearity analysis of A03 homeolog (39.00–44.60 Mb) among the ‘ZBC’, ‘AJ’, and ‘HS’ genomes. The 42.98–43.84 Mb interval in ‘ZBC’ had no collinear segments in either the ‘AJ’ or ‘HS’ genomes. The lower read coverage along the same A03 chromosome region after mapping resequencing data from ZBC-1 and W01-26 to the ‘ZBC’ genome. The significant increase in read coverage beyond 43.84 Mb is due to the higher distribution of *Gypsy* LTR-RTs in this region. (C) Evolutionary analysis identified 48 homologous genes in the collinear regions of ‘ZBC’, *B. carinata*, *B. juncea*, and *B. nigra*. *Ks* values were calculated by comparing the homologs of *B. juncea*, *B. carinata*, and *B. nigra* with those of ‘ZBC’. (D) Phylogenetic analysis based on 48 gene orthologs from collinear regions of ‘ZBC’ (BraA03), *B. carinata* (BcaB05), *B. juncea* (BjuB05), and *B. nigra* (BniB05). The 48 homologous proteins from four species were aligned, truncated, and merged into a single sequence to construct a phylogenetic tree.

Notably, an ~854.53-kb fragment on the short arm of chromosome A03 (between 42.98 and 43.84 Mb) in the ‘ZBC’ genome was not identified to be part of a collinear block in either the ‘AJ’ or ‘HS’ genomes. This suggests that this fragment may be unique to the ‘ZBC’ genome (Fig. 3B). Additionally, when we mapped the resequencing data from ZBC-1 and W01-26 to the ‘ZBC’ genome, we observed that read coverage in the 42.98–43.84 Mb region in W01-26 was significantly lower than that of other regions due to the high abundance of *Gypsy* LTR-RTs in this region. This further supports the idea that this fragment is unique to the ‘ZBC’ genome (Fig. 3B). Additionally, we utilized the Integrative Genomics Viewer (IGV) (https://igv.org/) to assess the coverage of introgression breakpoints following the alignment of HiFi sequencing reads and Illumina reads to the ‘ZBC’ genome. Furthermore, we designed specific markers to validate the accuracy of both the location and length of the 854.53-kb introgression fragment (Figs S8 and S9; Table S14). Evolutionary analysis revealed that the median *Ks* values of collinear genes within the ‘ZBC’ introgressed fragment were comparable to those found within the homeologous regions in *B. nigra*, *B. carinata*, and *B. juncea* (Fig. 3C**;**  Table S15). We subsequently aligned the fragment sequence with the genomes of six *Brassica* species, concluding that it is shared by *B. nigra* (BB genome) and the B-genome carrying allotetraploid species (*B. carinata*: BBCC genome, *B. juncea*: AABB) (Figs S10 and S11). Phylogenetic analysis of these collinear genes indicated that the introgressed fragment in ‘ZBC’ is more closely related to *B. carinata*, suggesting that it likely originated from *B. carinata* (Fig. 3D).

### The purple leaf trait in ‘ZBC’ is associated with the expression of *BraMYB114*

To identify the key gene regulating the purple leaf trait in ‘ZBC’, we performed reciprocal crosses between ZBC-1 (Fig. 4A) and W01-26 (Fig. 4B). The resulting hybrids displayed pale purple leaves, indicating that the purple leaf trait is not influenced by cytoplasmic genetic effects (Fig. 4C and D). The hybrids were self-pollinated and backcrossed with W01-26 to generate F_2_ segregating population and BC_1_F_1_ backcross segregating population. Among the 485 F_2_ segregating individuals, 358 exhibited purple leaves, and 127 had green leaves, resulting in a segregation ratio of ~3:1 (χ^2^ = 0.43). In the 346 BC_1_F_1_ backcross segregating population, 165 plants displayed purple leaves, and 181 had green leaves, yielding a segregation ratio of ~1:1 (χ^2^ = 0.65). These results suggest that the purple leaf trait in ‘ZBC’ is governed by an incompletely dominant locus. Subsequently, we conducted BSA-seq analysis using extreme green and purple individuals from the F_2_-segregating population. The analysis revealed a significant peak of indels between 24.72 and 44.28 Mb on chromosome A03 in the ‘ZBC’ genome (Fig. 4E and F). Further analysis indicated that the centromeric and pericentromeric regions of chromosome A03 fall within the 34.08–39.38 Mb interval (Fig. 4G). Since (peri)centromeric regions are highly repetitive, this divides the target interval into two smaller regions on either side of the centromere (24.72–34.08 Mb and 39.38–44.28 Mb) (Fig. 4G). Previously, we mapped the gene *BraANS.A03* between 24.72 and 34.08 Mb on the long arm of chromosome A03 [31]. However, the *BraCHI* gene is also located in this region [32]. Based on these phenotypes, results from BSA-seq, and fine mapping results, we assumed that another key gene for the purple leaf trait may reside in the interval between 39.38 and 44.28 Mb on the short arm of chromosome A03. This gene may not have been identified in previous studies.

**Figure 4:**
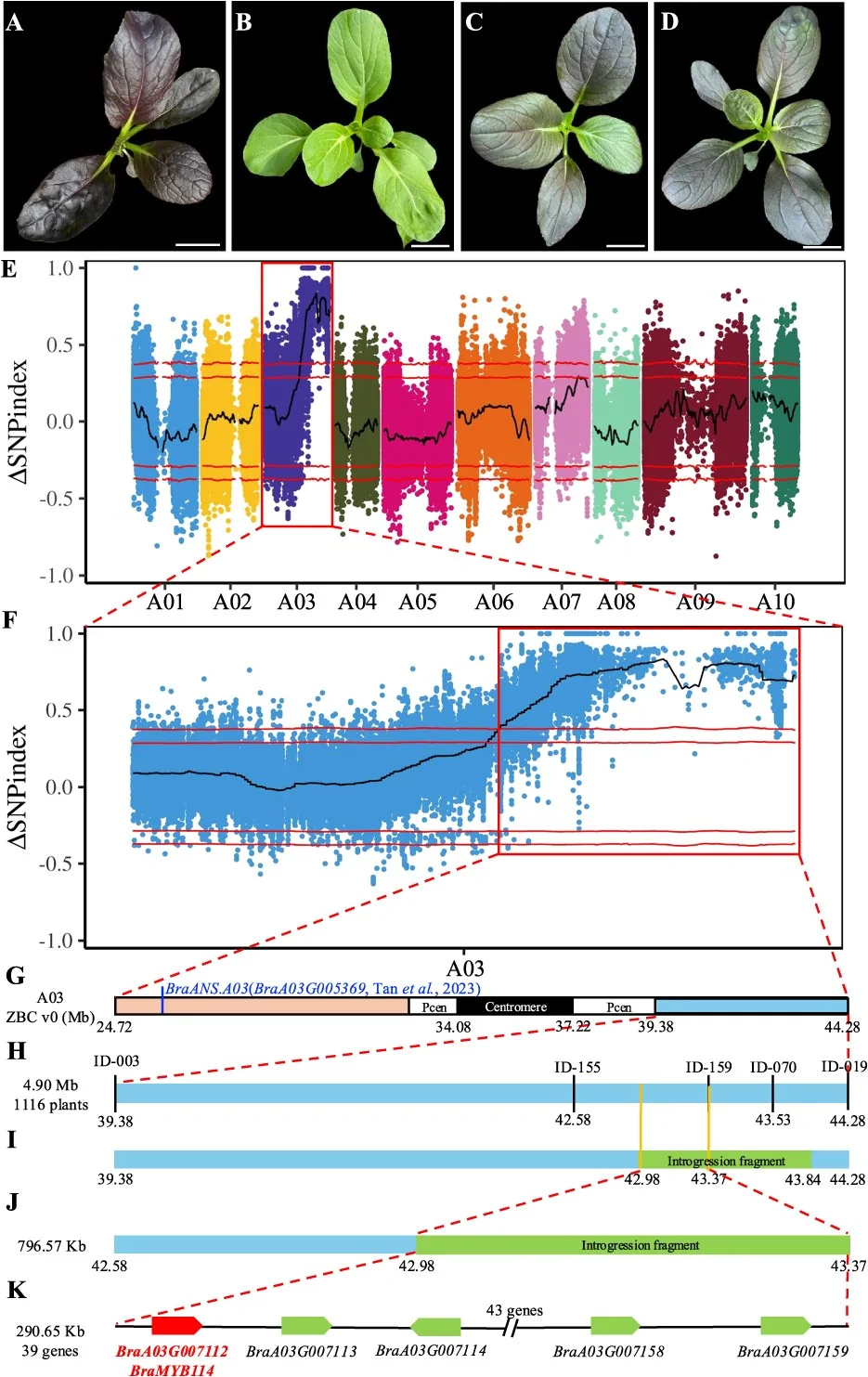
Fine mapping of the *BraMYB114* gene in ‘ZBC’. (A–D) The phenotypes of ZBC-1, the hybrid F_1_ plants (♀ZBC-1 × W01–26♂, ♀W01–26 × ZBC-1♂), and W01-26. Scale bars: 2 cm. (E) BSA-seq analysis to identify candidate intervals associated with the purple leaf trait. The four red lines in the *x*-axis represent CI values of 0.95 (inner) and 0.99 (outer), respectively. The ΔSNP-index value on the *y*-axis is derived by calculating the Δ distribution, taking into account the proportion of alleles from the purple and green pools relative to the parental ZBC-1. (F) The candidate region mapped to chromosome A03 (24.72–44.28 Mb). (G) The 19.56-Mb candidate region includes the centromere and adjacent pericentromeric regions. Pcen: pericentromere. *BraA03G005369* corresponds to the genome number of the *BraANS.A03* locus gene in the ZBC genome, whereas *BraA03g050560.3C* represents the genome number for the Chiifu genome (v3.0) [31]. (H) Fine mapping of the purple leaf trait gene to the short arm of A03. A total of 1116 extreme loser plants were used for fine mapping of the purple-leaf trait gene, based on the map-based cloning method, the target region was finally locked between ID155 and ID159. ID: insert or delete marker. (I) The 854.53-kb introgression fragment from *B. carinata* on the short arm of A03. The results of the introgression fragments are derived from the previous genomic variation analysis in this study (see Fig. 3 for details). The chromosomal regions between the yellow bars represent the overlapping areas between the introgression fragments and the gene mapping regions for the purple leaf trait. (J) The introgression fragment overlaps with the target region at 42.98–43.37 Mb, suggesting that the purple leaf trait gene of ‘ZBC’ located in this region. (K) The candidate gene prediction. *MYB114*-like transcription factor *BraA03G007112* regulates the purple leaf traits of ‘ZBC’.

To further verify our hypothesis, a total of 1116 green leaf plants from the F_2_-segregating population were selected for fine mapping the purple leaf trait in ‘ZBC’. We initially designed 10 pairs of indel markers targeting the region at 39.38 and 44.28 Mb. Ultimately, we selected markers InDel003 and InDel019 to screen for plants exhibiting chromosomal segment exchange within the target interval. We then employed chromosome walking to narrow down the target interval. This method allows us to extend the target region to InDel155 near the centromere and to InDel159 near the telomere, containing 12 and 13 plants with chromosome segment exchanges in the target region, respectively. The distance between InDel155 and InDel159 was 796.57 kb, and we were unable to narrow it further due to the lack of additional indel markers (Fig. 4H). Notably, the introgressed fragment identified in the variation analysis was 854.53 kb in length, located between 42.98 and 43.84 Mb, and overlapping with the target interval. This introgression may explain the difficulties encountered in narrowing down the target region for the fine mapping of the purple leaf trait gene. The recombination and exchange of chromosome segments in this region are inherently difficult (Fig. 4I). Consequently, we hypothesize that the purple leaf trait gene is likely located within the overlapping region (42.98–43.84 Mb) between the introgressed fragment and the target interval (Fig. 4J). Gene annotation indicated that this overlapped fragment contains 48 genes, of which *BraA03G007112* and *BraA03G007147* are implicated in leaf color regulation (Table S16). *BraA03G007112* is a *MYB114*-like transcription factor, while *BraA03G007147* encodes a photosystem II core complex protein, psbY, which is involved in regulating chlorophyll synthesis (Table S16). Therefore, we speculated that *BraA03G007112* is the key gene responsible for the purple leaf trait in ‘ZBC’, and we have named it *BraMYB114* (Fig. 4K).

Analysis of chromosomal variation indicated that the introgressed fragment containing *BraMYB114* likely originated from the B subgenome (Figs S10 and S11). To further validate these results, we identified *BraMYB114* orthologs across six *Brassica* species, yielding a total of 35 orthologs (Table S17). Notably, phylogenetic analysis revealed that these genes can be classified into four clades. *BraMYB114* clustered with *BjuB05g10780S* from *B. juncea* and *BcaB05g24263* from *B. carinata*. Specifically, *BraMYB114* and *BcaB05g24263* are within the same subclade, indicating their close evolutionary relationship (Fig. S12). This further supports the conclusion that the introgressed fragment originates from *B. carinata*.

### Anthocyanin biosynthesis in ‘ZBC’

To analyze the regulatory mechanisms of anthocyanin biosynthesis in ‘ZBC’, we examined the total anthocyanin content in the leaves of two varieties: ZBC-1 and W01-26. The results indicated that the anthocyanin content in ZBC-1 leaves was 0.59 μg/g of flesh weight (FW), while 0.02 μg/g FW were estimated in W01-26 leaves (Fig. 5A). We also conducted RNA-seq and metabolomic analysis of the leaves from both ZBC-1 and W01-26. The anthocyanin metabolome analysis identified 54 different anthocyanin metabolites, which included 3 types of petunidins, 6 types of delphinidins, 1 type of flavonoid, 1 type of malvidin, 7 types of peonidins, 27 types of cyanidins, and 9 types of pelargonidins (Table S18). Differential accumulation metabolites (DAMs) analysis revealed that 42 types of anthocyanins at higher levels in ZBC-1 leaves, whereas 12 types were lower (Fig. 5B). In W01-26, only Anthocyanidin_41 (Quercetin-3-O-glucoside) and Anthocyanidin_371 (Petunidin-3-O-(6″-O-malonyl)-glucoside-5-O-glucoside) were detected at levels ≥1 μg/g dry weight (DW). Conversely, in ZBC-1, the levels of 12 anthocyanin metabolites were ≥1 μg/g DW, with Anthocyanidin_41 (Quercetin-3-O-glucoside), Anthocyanidin_148 (Cyanidin-3-O-hydroxybenzoyl-xyloside-5-malonyl-glucoside), and Anthocyanidin_145 (Cyanidin-caffeoyl-glucoside-glucoside) being the most abundant (Fig. 5E; Table S18).

**Figure 5:**
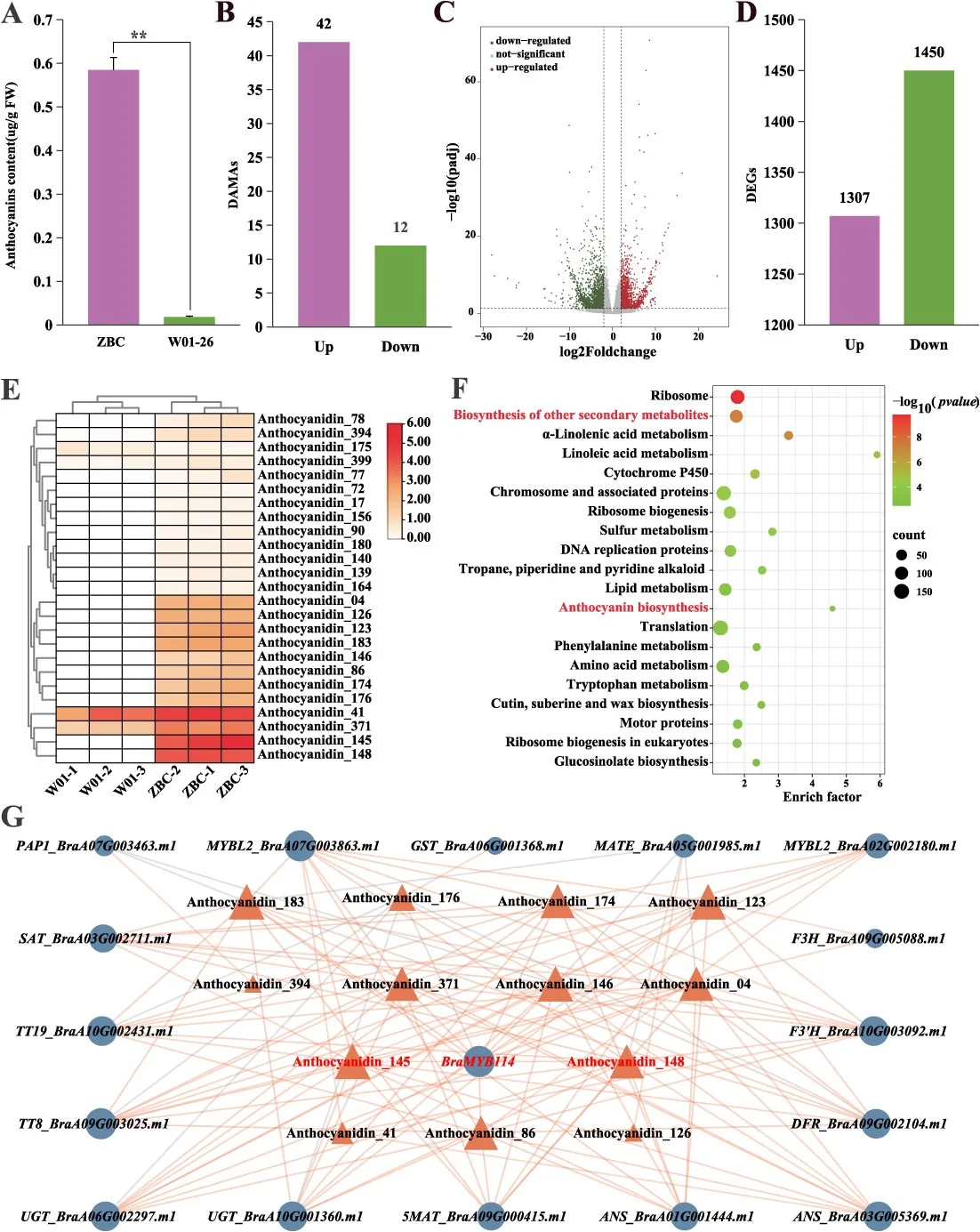
Metabolome and transcriptome analysis of ZBC-1 and W01-26. **(**A) Total anthocyanin content. ** means *P* < 0.01 of Student’s *t*-test. (B) Analysis of differentially accumulated metabolites (DAMs) between ZBC-1 and W01-26 leaves. (C, D) Analysis of differentially expressed genes (DEGs) between ZBC-1 and W01-26 leaves. (E) A clustered heat map of anthocyanins content (≥1 μg/g dry weight) in ZBC-1 and W01-26 leaves. The heatmap was plotted after taking the log_2_ values of all metabolites. (F) KEGG enrichment analysis of differentially expressed genes (DEGs) between ZBC-1 and W01-26 leaves. The details are listed in Table S21. (G) The regulatory network of DAMs and DEGs. Blue circles represent ABGs, orange triangles indicate DAMs, orange lines denote a positive correlation, and gray lines indicate a negative correlation. The hub gene (*BraMYB114*) and metabolites (Anthocyanidin_145 and Anthocyanidin_148) are highlighted in red.

RNA-seq analysis identified 2757 differentially expressed genes (DEGs) between the leaves of ZBC-1 and W01-26. Among these, 1307 DEGs were upregulated in ZBC-1, while 1450 DEGs were upregulated in W01-26 (Fig. 5C and D; Table S19). We subsequently conducted GO and KEGG enrichment analyses on these 2757 DEGs (Tables S20 and S21). The gene ontology (GO) analysis indicated that these genes were primarily involved in pathways related to stress response, stimulus response, and secondary metabolic processes (Fig. S13; Table S20). The KEGG analysis revealed significant enrichment of these genes in pathways associated with metabolism, anthocyanin biosynthesis, and the biosynthesis of other secondary metabolites. This suggests that these genes play a crucial role in the transcriptional regulation of metabolites, particularly anthocyanins (Fig. 5F; Table S21). Notably, the DEGs were also significantly enriched in pathways related to α-linolenic acid and linoleic acid metabolism. This observation aligns with the KEGG results for the duplicated genes of PD- and TD-types. As α-linolenic acid and linoleic acid are essential fatty acids crucial for human health, these results suggest that the ‘ZBC’ variety may have a higher nutritional value compared to other varieties of *B. rapa*. In addition, an analysis was conducted to investigate the relationship between the expression profiles of anthocyanin biosynthesis genes (ABGs) in ZBC-1 and W01-26 and their respective anthocyanin metabolites. The results demonstrated that *BraMYB114* was positively correlated with the metabolism of 13 distinct anthocyanins (Fig. 5G; Table S22). These findings further highlight the important role of *BraMYB114* in the transcriptional regulation of anthocyanins in the ‘ZBC’ variety.

### *BraMYB114* is a key transcription factor regulating anthocyanin biosynthesis in *B. rapa*

We further identified genes involved in the anthocyanin biosynthesis pathway within the ‘ZBC’ genome and constructed a heatmap to illustrate the gene expression associated with this pathway. This analysis was based on RNA-seq expression data from the leaves of ZBC-1 and W01-26 (Fig. 6A; Table S23). The genes related to the phenylpropanoid biosynthesis pathway, such as *PAL*, *C4H*, and *4CL*, did not show distinct differences in expression patterns between ZBC-1 and W01-26, likely due to their involvement in multiple metabolic pathways. In contrast, genes in the flavonoid biosynthesis pathway, including *CHS*, *CHI*, *F3H*, and *F3′H*, exhibited significantly higher expression levels in ZBC-1 compared to W01-26. Notably, flavonol synthase (FLS), which is essential for flavonol biosynthesis, showed markedly higher expression in W01-26. Additionally, *DFR* and *ANS*, both essential for anthocyanin catalysis, were expressed at significantly higher levels in ZBC-1. The modification proteins SAT and 5MAT were highly expressed in ZBC-1, whereas *OMT1* and *MATE* were predominantly expressed in W01-26. The expression patterns of different copies of *UGT*, *SGT*, and *GST* varied between the two accessions. The anthocyanin transporter *TT19* was highly expressed in ZBC-1. Interestingly, all activators, with the exceptions of *BraMYB114* and *TT8*, were highly expressed in W01-26. In contrast, the repressors *CPC*, *MYBL2*, and *LBD* showed markedly higher expression levels in ZBC-1 (Fig. 6A).

**Figure 6:**
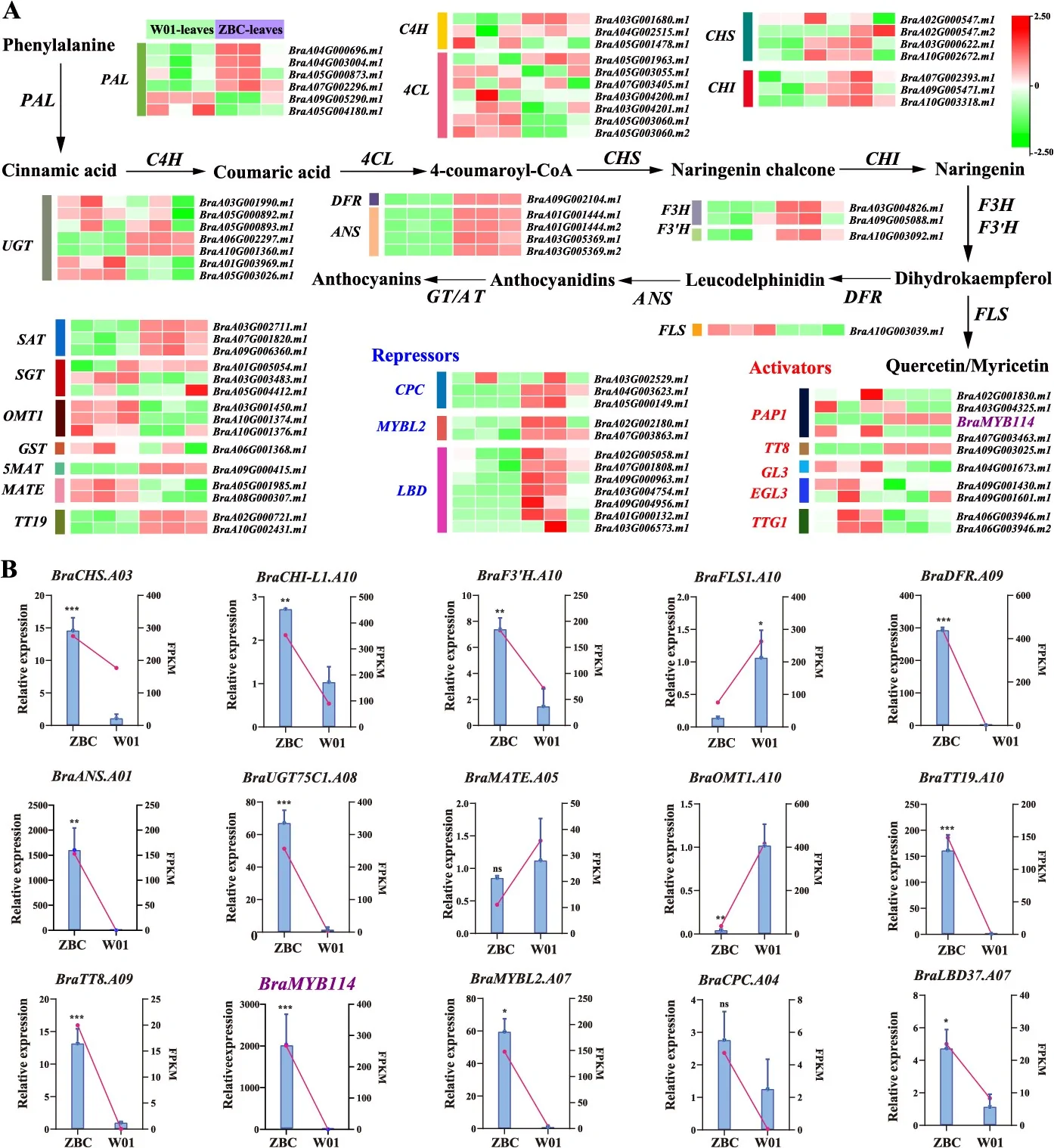
Expression patterns of ABGs in ZBC-1 and W01-26. (A) The expression pattern of ABGs in the leaves of ZBC-1 and W01-26. Genes marked in blue are repressors, while those marked in red are activators. The heatmap was generated using genes with an FPKM value ≥1. (B) The relative expression levels of ABGs in the leaves of ZBC-1 and W01-26. The blue bar graph represents the results of qRT-PCR analysis, while the red line indicates the FPKM values obtained from RNA-seq analysis. ^*^ means *P* < 0.05 ^**^ means *P* < 0.01, and ^***^ means *P* < 0.001 of Student’s *t*-test.

To further analyze the expression patterns of *BraMYB114* and its three homologs, we collected RNA-seq data from purple- and green-leaf Chinese cabbage [33], Wutacai (*B. rapa* subsp. *narinosa*) [31], and ‘ZBC’ [34], along with ZBC-1 and W01-26 in this research. The results showed that *BraMYB114* was expressed at very low levels in green-leaf varieties (W01-26, Wutacai, green-leaf Chinese cabbage), while it was highly expressed in purple-leaf varieties (ZBC-1 and green-leaf Chinese cabbage) (Fig. S14; Table S24). Furthermore, the three *BraMYB114* homologs (*BraA02G001830*, *BraA03G004325*, and *BraA07G003463*) were found to have low expression levels in the leaves of all accessions (Fig. S14; Table S25). These findings suggest that *BraMYB114* regulates anthocyanin biosynthesis in *B. rapa*.

We selected 15 DEGs from ABGs and designed specific markers for quantitative real-time PCR (qRT-PCR) analysis (Table S25). The results showed that 12 genes—*BraCHS.A03*, *BraCHI-L1.A10*, *BraF3′H.A10*, *BraDFR.A09*, *BraANS.A01*, *BraUGT75C1.A08*, *BraTT19.A10*, *BraMYB114*, *BraTT8.A09*, *BraMYBL2.A07*, *BraCPC.A04*, and *BraLBD37.A07*—were significantly more expressed in ZBC-1. In contrast, *BraFLS.A10*, *BraMATE.A05*, and *BraOMT.A10* exhibited higher expression levels in W01-26 (Fig. 6B). BraFLS.A10 is a key enzyme that catalyzes the biosynthesis of flavones, competing with BraF3*′*H.A10 for the substrate flavanones. BraMATE.A05, BraOMT.A10, and BraSAT.A03 are modifying enzymes, suggesting that flavones may undergo more extensive modifications. Specifically, *BraMYB114* in ZBC-1 regulates the expression of genes such as *BraDFR.A09*, leading to the predominant conversion of flavonoids into anthocyanins. However, in W01-26, BraFLS.A10 catalyzes the synthesis of quercetin or myricetin from flavonoids. Interestingly, the activators *BraMYB114* and *BraTT8.A09*, along with the repressors *BraMYBL2.A07*, *BraCPC.A04*, and *BraLBD37.A07*, were expressed at higher levels in ZBC-1 compared to W01-26. This observation likely indicates the activation of negative feedback regulation due to the abundant synthesis and accumulation of anthocyanins (Fig. 6B).

To investigate the biological function of *BraMYB114*, we constructed transgenic *A. thaliana* plants that overexpress this gene under the control of the 35S CaMV promoter (Fig. S15). The cotyledons and hypocotyls of these overexpressing plants exhibited a purple coloration, indicating that *BraMYB114* can activate the synthesis and accumulation of anthocyanins in *A. thaliana*. Then, to elucidate the regulatory pattern of the transcription factor *BraMYB114*, we confirmed the protein-level interaction between BraMYB114 and BraTT8 in the nuclei of tobacco (*Nicotiana benthamiana*) leaf epidermal cells using bimolecular fluorescence complementation (BiFC) technology (Fig. S16). Furthermore, to identify the downstream target genes regulated by *BraMYB114*, we cloned the promoter sequences of *BrANS.A01* and *BrDFR.A09* based on RNA-seq analysis results and performed yeast one-hybrid assays for validation. These assays further confirmed that BrMYB114 binds to the promoters of *BrANS.A01* and *BrDFR.A09* (Fig. S17).

Furthermore, we developed four pairs of specific molecular markers for *BraMYB114* (Fig. S18; Table S26). By utilizing F_2_-segregating populations of W01-26 and ZBC-1 (Fig. S19A), and validating these findings with 19 germplasm resources for in *B. rapa* (Fig. S19B), we discovered that these specific markers were completely linked to the purple leaf trait. These findings further support the hypothesis that the introgression event associated with *BraMYB114* is likely specific to purple pak choi. Consequently, these markers provide valuable assistance for future breeding efforts aimed at developing new cabbage varieties rich in anthocyanins.

## Discussion

*B. rapa* is known for its considerable diversity, including a range of forms such as fleshy roots, midribs, leaves, stalks, and oilseeds [3]. This species is cultivated worldwide as both a vegetable and an oilseed crop, playing a crucial role in human nutrition. Pak choi, commonly known as Shanghaiqin or Xiaobaicai in China, is especially valued by consumers for its diverse cultivars and nutrient-rich profile, including high levels of calcium, iron, and vitamins. The distinctive purple hue of the ‘ZBC’ variety is attributed to its high anthocyanin content, a bioactive antioxidant recognized for its potential health benefits. These benefits may include the prevention of cardiovascular and cerebrovascular diseases, as well as liver protection [35].

### Identification of the purple leaf trait in *B. rapa*

Common purple varieties of *B. rapa* include turnip, Zicaitai, Chinese cabbage, pak choi, and oilseeds. Researchers mapped the purple coloration locus, *anl*, to linkage group R9 using anthocyanin-free mutants of rapid-cycling *B. rapa*. This marked the first report of a gene associated with the purple leaf trait in the species [22]. Then, the first gene, *BrPur*, controlling the purple leaf color in nonheading Chinese cabbage, was located on linkage group A03 [23]. Advancements in sequencing technology have accelerated the discovery of genes responsible for purple coloration in various *B. rapa* varieties. Key identified genes include *BrTT8* [24, 25], *BrMYB2* [21, 25, 30], *bHLH49* [26], *BrMYB73* [27], *BrMYBL2.1* [28], *BrPAP1a* [29], *BraANS.A03* [31], and *BraCHI* [32]. *BrTT8* and *bHLH49* are bHLH transcription factors, while *BrMYB2*, *BrPAP1a*, and *BrMYBL2.1* are MYB transcription factors. *BraANS.A03* and *BraCHI* function as catalytic enzymes in anthocyanin biosynthesis, regulated by MYB transcription factors or MYB-bHLH-WD40 (MBW) transcription complexes, highlighting the role of transcription factors in the color variation observed in *B. rapa*.

He *et al*. [30] found that high expression of *BrMYB2* is crucial for anthocyanin biosynthesis in Zicaitai, with the short intron 1 promoting its expression while the long intron 1 inhibiting it. Zhou *et al*. [21] showed that a *Gypsy* LTR-RT inserted in its promoter region regulates its expression. Similar patterns were observed in *B. oleracea*, where variation in the *BoMYB2* promoter activated expression in three purple cabbage varieties: kale, kohlrabi, and cabbage [36]. The intron transposon insertion within the R2R3-MYB transcription factor *BjPur* interferes with anthocyanin accumulation in the leaf of *B. juncea* by inducing epigenetic modifications [37].

Here, we identified a novel gene, *BraMYB114*, introgressed from *B. carinata* into *B. rapa* (Fig. 4). Sequence analysis revealed that *BraMYB114*, like *BrMYB2* and *BoMYB2*, is a homolog of *MYB75/90/113/114,* activating anthocyanin biosynthesis, suggesting that the purple trait in *B. rapa* is the result of an acquired mutation. This is the first report of an introgressed gene regulating the formation of the purple trait in *B. rapa*. More importantly, it elucidates why the gene responsible for the purple leaf trait in pak choi has not been accurately fine-mapped over the past two decades. This discovery enhances our understanding of the genes regulating the purple leaf trait in *B. rapa*, providing new insights into plant color variation and cultivar selection. Notably, *BrMYBL2.1*, a known repressor of anthocyanin synthesis, a 100-bp insertion in its third exon that alters the coding sequence (CDS) in ‘Zicaitai’, potentially leading to the loss of its inhibitory function [28]. This observation suggests the coexistence of both activation and repression mechanisms in *B. rapa*. Moreover, RNA-seq and qRT-PCR analyses revealed elevated expression levels of *BraMYB114* and *BrMYBL2* in ZBC-1 (Fig. 6; Fig. S14). Consequently, we pose the following questions: Is the purple leaf trait in *B. rapa* attributable to the activation of genes such as *BrMYB2* and *BraMYB114*, or is it a consequence of the loss of inhibitors like *BrMYBL2.1*? Additionally, does the simultaneous high expression of *BraMYB114* and *BrMYBL2* in ZBC-1 suggest that *BraMYB114* may induce the expression of *BrMYBL2* through feedback regulation? These inquiries necessitate further investigation.

### Introgression enhances the genetic diversity of *B. rapa*

Distant hybridization is a crucial tool in plant breeding, particularly for acquiring resistance genes from closely related wild species. Previous studies have conducted numerous hybridizations with *B. carinata* [38, 39] and *B. napus* [40] to introduce superior agronomic traits into *B. rapa*. However, there are hardly any reports regarding the introgression of B subgenome fragments into *B. rapa* (AA). Here, we assembled a nearly gap-free, chromosome-level genome of ‘ZBC’ and identified an introgressed 854.53-kb fragment on chromosome A03 that likely originated from *B. carinata* (Fig. 4D). This fragment overlaps with a previously identified fine mapping target region by 390 kb and contains the *BraMYB114* gene, which plays a crucial role in regulating expression of the purple trait in ‘ZBC’ (Figs 5 and 6). The previously identified *BrMYB2* gene in Zicaitai primarily regulates the purple-red coloration of Chinese cabbage [21, 30]. Our research suggests that the introgressed fragment from *B. carinata* contributes to the regulation of purple leaf color in pak choi*. B. carinata* is recognized as an excellent source of genes for the genetic improvement of *Brassica* crops and numerous studies have documented the introgression of its genome into *B. napus* [41] and *B. juncea* [42]. However, there have been no reports of such introgression into *B. rapa*. Our research provides the first evidence of a *B. carinata* genomic fragment carrying *BraMYB114,* exhibiting a positive regulatory effect on anthocyanin biosynthesis, that has been introgressed into the *B. rapa* genome.

A similar observation has been documented in *B. napus*, where the introgression of the *BjMYB113* gene from *B. juncea* resulted in the purple leaf coloration [43]. Moreover, Tu *et al*. [44] identified an introgressed fragment from Chinese woad (*Isatis indigotica*) in 20 plants that resulted from self-pollination of distant hybridization between *B. rapa* and Chinese woad. In natural conditions, *B. napus*, which harbors a glyphosate-resistance gene, may also transfer glyphosate resistance horizontally to *B. rapa* through hybridization, introgression, and gradual chromosome loss [45]. Pandian *et al*. [46] successfully introgressed the herbicide resistance gene from *B. napus* into *B. rapa* via hybridization and backcrossing. Furthermore, allotriploid hybrids (AAC) produced by hybridizing autotetraploid *B. rapa* (AAAA) with *B. oleracea* (CC) can form trivalents during meiosis, which enhances the rate of introgression from the C genome into the A genome [47]. These results imply that both natural and artificial hybridization can facilitate the introgression of genes or chromosome fragments. The selective self-incompatibility of *B. rapa* promotes hybridization with closely related species, leading to the formation of interspecific hybrids and the subsequent fixation of introgressed lines. The continuous integration of exogenous genes may have contributed to the rich variation observed in *B. rapa* varieties.

## Conclusion

In summary, we assembled a nearly complete T2T genome of the ‘ZBC’. We identified an 854.53-kb introgression fragment from *B. carinata* located on chromosome A03. Fine mapping revealed that the *BraMYB114* on the introgression fragment may play a crucial role in controlling the purple leaf trait in ‘ZBC’. Transcriptomic, metabolomic, and qRT-PCR analyses demonstrated that *BraMYB114* positively regulates anthocyanin biosynthesis. Our study provided the first evidence that the *BrMYB114* gene present in the introgression fragment positively influences anthocyanin synthesis in *B. rapa*. These findings enhance our understanding of the *B. rapa* genome, introduce methodologies for studying key trait genes in introgression lines, and offer a unique resource for research on the genetics and evolution of *B. rapa*.

## Materials and methods

### Plant materials

A high-generation inbred line of ‘ZBC’ (purple leaf, ZBC-1, Fig. 1A) was used for whole-genome sequencing. The reciprocal hybrid F_1_ plants were obtained by reciprocal crossing ZBC-1 with another purebred anthocyanin-free line, Caixin (green leaf, *B. rapa* subsp. *parachinensis*, W01-26). The F_2_ and BC_1_F_1_ generation–segregating populations were obtained by self-crossing and backcrossing of F_1_. All plants were grown from seeds and cultivated in a greenhouse at the Gannan Normal University, Ganzhou, China.

### Sample collection and sequencing

Fresh leaves of the ‘ZBC’ were promptly collected from 7-leaf plants and immediately frozen in liquid nitrogen. The extracted genomic DNA was utilized for PacBio HiFi sequencing using the Sequel II system, as well as for Hi-C and Illumina sequencing on the NovaSeq 6000 platform. Tissues from seedlings (7 days after germination), leaves (7-leaf stage), roots (7-leaf stage), stems (during the bolting period), inflorescences, young siliques (3 weeks after blooming), and mature seeds were collected, promptly frozen in liquid nitrogen and stored at −80°C. Subsequently, RNA was isolated and sequenced using the NovaSeq 6000 platform for genome annotation purposes.

### Genome survey, assembly, and assessment

To *de novo* assemble the ‘ZBC’ genome, the genome size was initially estimated using GenomeScope v2.0 [48]. Subsequently, the PacBio HiFi data were assembled using hifiasm software v0.16.1 [49] to generate the assembly, followed by three rounds of correction utilizing minimap2 v2.17 [50] and Racon v1.4.19 [51] with PacBio HiFi long reads. Then, Illumina sequence data were analyzed using Burrows–Wheeler Alignment (BWA) v0.7.19-r1273 [52] for error correction, and the redundant sequences were filtered using Purge Haplotigs (v1.0.4) [53] with parameters -g genome.fasta; -c coverage_stats.csv. The pseudo-chromosomes were constructed using Hi-C data through the Juicer v2.0 [54] and 3D-DNA v180922 [55]. The binary files were generated using Juciertools v3.0 [54] and subsequently manually corrected with Juicebox v2.15.07 [56]. The quality of the assembled genome was evaluated using BUSCO v5.6.1 [57]. Subsequently, LTR-RTs within the genome assembly were identified and annotated using LTR_FINDER v1.07 [58] and LTRharvest v1.5.10 [59]. These results were then integrated, and the LAI was calculated using LTR_retriever v2.9.0 [60, 61].

### Genome annotation

Firstly, The TeloExplorer and CentroMiner module of quarTeT toolkit v1.2.4 [62] were used to predict telomere and centromere regions. Then, repetitive sequences and TEs were identified using the EDTA pipeline v2.2.2 [63] and DeepTE software v3.7.1 [64].

The *de novo* prediction was conducted using Augustus v3.3.3 [65] and GlimmerHMM v3.0.4 [66], with PacBio Iso-seq (RNA-seq) dataset. Subsequently, we employed GeMoMa software v1.9 [67] for homologous gene prediction, using protein sequences from *B. rapa* (‘Chiifu’ v4.0, ‘HS’, ‘AJ’, and ‘Z1’ v2.0), *B. napus* (‘ZS11’ v10), and *B. ju*ncea (‘varuna’ v0) as homologous gene sequences (Table S7). Transcript assembly was performed using StringTie v2.1.3 [68], and CDSs were predicted using TransDecoder v5.1.0 (https://github.com/TransDecoder/). Finally, the annotation results were integrated using EvidenceModeler v1.1.1 and PASA pipeline v2.5.2 [69]. Subsequently, the prediction of ribosomal RNA (rRNA), transfer RNA (tRNA), and noncoding RNA was performed using Barrnap v0.9 (https://github.com/tseemann/barrnap), tRNAscan v2.0.0 [70], and Infernal v1.1.3 (https://github.com/EddyRivasLab/infernal), respectively.

Protein prediction was performed by querying the NR and UniProt protein databases using Diamond v2.0.7.145 [71]. Subsequently, supplementary information was obtained using KAAS [72] and eggNOG-mapper v2 [73]. Protein domains were then annotated using HMMER v3.3.1 [74]. The details are consistent with those previously described [75, 76].

### Comparative genomic analysis

To identify common gene families among *B. rapa* (Chiifu v4.0, ECD04, ‘HS’, ‘AJ’, and Z1 v2.0), *B. oleracea* (HDEM), *B. nigra* (Ni100 LR v2.0), *Raphanus sativus*, *A. thaliana* (col-0, TAIR 10), *Orychophragmus violaceus*, *Erysimum cheiri*, *Matthiola incana*, and *Aethionema arabicum*, we used OrthoFinder software v2.5.2 [77] and Diamond were used to identify single-copy orthologous genes (Table S11). The sequences were then aligned using MUSCLE v3.8.31 [78] and phylogenetic relationships were estimated with RAxML software v8.2.12 [79]. Divergence times were estimated using the MCMCTree v4.9 program [80], with time calibration points obtained from the TimeTree website (http://www.timetree.org/).

We used CAFE v4.2.1 [15] to identify gene family contraction and expansion (Table S11). WGDI v0.6.5 [81] was used to identify collinear gene pairs, and then, multiple sequence alignment was performed using MUSCLE. KaKs_Calculator software v3.0 [82] was used to estimate nonsynonymous (*Ka*) and synonymous (*Ks*) substitution rates, the R package ggplot2 was used for plotting.

Genome-wide structural and sequences variations between ‘ZBC’, ‘AJ’, and ‘HS’ were aligned and detected using MUMmer v 4.0.1 [83] and SyRI [84]. We utilized BWA software to align the resequencing data from ZBC-1 and W01-26 to the ‘ZBC’ genome using default parameters, subsequently calculating the reads coverage for the interval of 42.98–44.60 Mb. This analysis confirmed that the fragment was unique to the ‘ZBC’ genome. Additionally, we used minimap2 to trace the origin of the introgressed fragment through whole-genome alignment, establishing that it originated from a *Brassica* species possessing the B subgenome (*B. juncea* (Wuqi), *B. carinata* (zd-1), and *B. nigra* (Ni100 LR v2.0)). Furthermore, we applied MUSCLE to identify shared orthologous genes among the homologous fragments across the four species and constructed a phylogenetic tree using RAxML software.

### Fine mapping of purple leaf trait gene

To locate the purple leaf trait gene of ‘ZBC’, the parents ZBC-1 and W01-26, 30 extreme purple and green leaves in the F_2_ plants were collected for BSA-seq. The extreme purple and green DNA mix pools were obtained by adding equal quantities of DNA from 30 extreme purple F_2_ plants (purple-pool) and 30 green F_2_ individuals (green-pool), along with two parents for BSA-seq.

The raw data of four pools (ZBC, ~78× genome coverage; W01, ~31×; purple-pool, ~90×; green-pool, ~82×) were purified using fastp software v 0.23.0 [85]. The clean data were aligned to ‘ZBC’ reference genome using BWA software. Subsequently, variant information was identified using GATK (v4.0.9.0) [86]. The GATK software requires a sequencing depth of ≥5× for both parents, a pooling depth of ≥10× for the progeny, all parents to be homozygous, to have interparental polymorphism, and a pooled SNP index value of ≥0.8 or ≤ 0.2. We used QTLseqr v.0.7.5.2 [87] to calculate the Δ(SNP-index) value. The data was processed using Python pipeline, and the Manhattan plot was generated utilizing R package (v3.6.2) ggplot2 (v3.3.2) (https://ggplot2.tidyverse.org/). The fitted ΔSNPindex values were sorted in descending order, and the top 1% of sites with the highest fitted Δ(SNPindex) values were identified as trait-related sites. Subsequently, these sites were concatenated based on their size; if the distance between any two sites was <1 Mb, they were merged into a single interval. Additionally, QTLseqr was choused to calculate the 95% and 99% confidence intervals (CIs).

Additionally, the leaves of 1116 green plants from the F_2_-segregating population were collected and utilized for map-based cloning to fine-map the key gene responsible for purple leaf of ‘ZBC’.

### Detection of anthocyanins

The total anthocyanin content in ZBC-1 and W01-26 fresh leaves was determined according to Chen *et al*. [88], each with six biological replicates. Then, the fresh leaves of ZBC-1 and W01-26 were collected, immediately frozen with liquid nitrogen and stored at −80°C for metabolome analysis. The qualitative and quantitative analysis of anthocyanins were detected by Metware Biotechnology Co., Ltd. (Wuhan, China).

### RNA sequencing and transcriptome analysis

Young leaves of the 7-leaf stage for ZBC-1 and W01-26 plants, with three biological replicates, were collected for RNA-seq on the Illumina HiSeq NovaSeq 6000 platform. We also used RNA-seq data of WTC leaves (PRJNA881503) and RNA-seq data at four developmental stages of ‘ZBC’ seeding stage (PRJNA1146938) obtained from our previous research, and the RNA-seq data at the 4-leaf, 6-leaf, and 10-leaf stages of Chinese cabbage leaves were collected (PRJNA770000) to analyze the expression patterns of *BraMYB114* and its three homologous copies in the varieties of *B. rapa*. The RNA-seq data analysis method refers to Chen *et al*. [75].

### Quantitative analysis of key genes in anthocyanins biosynthesis pathway

Total RNA isolated and first-strand cDNA production of ZBC-1 and W01-26 was as described above, each sample with three biological replicates. Gene-specific primers were designed using DNAMAN software v10 (https://www.lynnon.com/). We used *Actin3* as an internal reference gene for qRT-PCR, and the relative gene expression was calculated according to 2^-ΔΔ^CT method. The detailed qRT-PCR experimental procedure and data analysis methods were described earlier [75, 88], and primer information is listed in Table S24. *BraMYB114* specific primers were designed using DNAMAN software, and primer information is listed in Table S25.

### Vector construction and transformation of *A. thaliana*

To construct the overexpression vector *35S:BraMYB114*, we amplified the CDS from the cDNA of ‘ZBC’. The polymerase chain reaction (PCR product was cloned into the plant expression vector pBI121 (fused with a GFP tag), and the construct was stably transformed into *A. thaliana* (Col-0) via *Agrobacterium*-mediated transformation.

### BiFC verification

In our BiFC experiments, we utilized expression vectors pSPYCE- BraMYB114 and pSPYNE-BraTT8, which encode the N-terminal and C-terminal fragments of yellow fluorescent protein, respectively. The CDS sequence of *BraMYB114* was cloned into the pSPYCE vector to create BraMYB114-cYFP, while the CDS sequence of *BraTT8* (*BraA09G003025*) was inserted into the pSPYNE vector to produce BraTT8-nYFP, respectively. These constructed vectors were subsequently transformed into the leaves of *N. benthamiana* for transient expression analysis. The transfected cells were then imaged using a confocal microscope (TCS SP8; Leica).

### Y1H analysis

For the Y1H assays, the CDS of *BraMYB114* was inserted into the pGADT7 vector to generate the *BraMYB114*-AD construct. The promoters of *BraDFR.A09* and *BraANS.A01* were subsequently inserted into the pHis2 vector, resulting in the pro*BraDFR.A09*-pHis2 and pro*BraANS.A01*-pHis2 constructs. Y1H assays were conducted using the Yeastmaker Yeast Transformation System (Clontech, USA). The corresponding vectors were co-transformed into the yeast strain Y187. Self-activation was inhibited by 3-amino-1,2,4-triazole (3-AT) (Coolaber, Beijing, China). Yeast cells were cultivated at 30°C for a duration of 3 days.

### Statistical analysis

Student’s *t*-test was employed for statistical comparisons between two groups. To investigate the differences in the distribution density of *Gypsy*- and *Copia*-type LTR-RTs across the chromosome arms, centromeres, and pericentromeres within the ‘ZBC’ genome, we first established the centromere region based on identification results. The region exhibiting a *Gypsy*-type LTR-RTs distribution >0.1 (with 100-kb window), extending up to the centromere, was classified as pericentromeres, while the remaining segments were designated as chromosome arms (Tables S27 and S28).

## Supplementary Material

Web_Material_uhag193

## Data Availability

The raw sequencing data and genome assemblies reported in this study are accessible through National Genomics Data Center (https://www.cncb.ac.cn/) under the BioProject identifier PRJCA048442. We also collected RNA-seq from NCBI (https://www.ncbi.nlm.nih.gov/) with BioProject identifier PRJNA881503, PRJNA1146938, and PRJNA770000.
