# Supplementary material for: Near telomere-to-telomere genome assembly enables the discovery of introgressed purple-leaf gene BraMYB114 in pak choi (Brassica rapa subsp. chinensis)
Source: Hortic Res. 2026 May 13;13(10):uhag193. doi: 10.1093/hr/uhag193 (PMC13623495; doi:10.1093/hr/uhag193)
Supplement: Supplementary file 1 [file web_material_uhag193.zip › Suppl figures 05 April 26_MAL.docx]

**Supplementary Figures**

**Near telomere-to-telomere genome assembly enables the discovery of introgressed purple-leaf gene *BraMYB114* in pak choi (*Brassica rapa* subsp. *chinensis*)**

Chen *et al.*

**Figure S1 Analysis of the *B. rapa* genome size based on a *k-mer* analysis.** The *k-mer* frequency distribution (*k* = 17) indicates that the estimated genome size of *B. rapa* is 456.48 Mb.

**Figure S2 The distribution of repetitive sequences and the locations of gaps, centromeres, and telomeres in the “ZBC” genome.** The repetitive sequences were analyzed using the quarTeT software, including information on gaps, telomeres, and centromeres. The details are list in Suppl. Tables S3-5.

**Figure S3 The heatmap of interactions of centromeric satellite repeats in the centromere regions of the 10 chromosomes of the ‘ZBC’ genome.**

**Figure S4 The transposable elements density in the ‘ZBC’ genome.** Windows size 100 Kb.

**Figure S5 The *Gypsy-* (A) and *Copia-*type (B) LTR-RTs density in chromosome arms，centromeres and pericentromeres of ‘ZBC’ genome.** Windows size 100 Kb. Differences between chromosome arms, centromeric regions and pericentromeric regions were assessed using

Student's *t*-test.

**Figure S6 The phylogenetic relationships and divergence time estimates for the ZBC variety and the other 13 Brassicaceae taxa.** The 1,868 shared single-copy orthologous genes were used to construct the phylogenetic tree. The red and blue numbers quantify the expansion/contraction of gene families; numbers in black show estimated divergence times.

**Figure S7 KEGG enrichment analysis of 539 expanded gene families**. The genes are listed in Suppl. Table S12. The pathways related to plant secondary metabolite biosynthesis and stress resistance are highlighted in red.

**Figure S8 The location of left (A) and right (B) breakpoints and HiFi sequencing reads distribution in the introgressed fragment in ‘ZBC’ genome.**

**Figure S9 Detection of left and right breakpoints of introgressed fragment using specific primers.** Markers L400 and R400 are the markers for the left and right breakpoints of introgressed fragment, respectively.

**Figure S10 An 854.53-kb fragment on chromosome A03 in ZBC (x axis) was identified as colinear genomic blocks in *B. juncea* (Varuna v0)*, B. carinata*** **(zd-1 v0), and *B. nigra* (Ni100 v2.0) (y axis).**

**Figure S11 Gene colinearity diagram of the ZBC 854.53 kb introgressed fragment and homeologous regions in *B. juncea* (17.24 Mb) (Varuna v0), *B. carinata* (17.64 Mb) (zd-1 v0), and *B. nigra* (15.38 Mb) (Ni100 v2.0).** The red ribbons connect collinear genes.

**Figure S12 Phylogenetic analysis of *BraMYB114* homologous genes in *Arabidopsis thaliana* and *Brassica* species**. The genes are listed in Suppl. Table S16. *BraMYB114* is highlighted in bold blue. AT: *A. thaliana*; Bra: *Brassica rapa* (Chiifu); Bol: *B. oleracea* (HDEM); Bni: *B. nigra* (Ni100); Bju: *B. juncea* (Varuna); Bca: *B. carinata* (zd-1); BnaDAR: *B. napus* (Darmor).

**Figure S13 Gene Ontology (GO) enrichment analysis of differentially expressed genes (DEGs) between ZBC-1 and W01-26.** BP: biological process; CC: cellular component;; MF: molecular function.

**Figure S14 Expression patterns of *BraMYB114* and its three homologous copies.** W01: W01-26; WTC: Wutacai; S0-S3: the four developmental stages of ‘ZBC-1’ during the cotyledon stage; GC-C, GC-I and GC-II: the three developmental stages (the cotyledon stage about four true leaves, seedling stage about six true leaves, and large-leaf stage about 10 true leaves) of green-leaf Chinese cabbage; PC-C, PC-I, and PC-II: the three developmental stages (the cotyledon stage about four true leaves, seedling stage about six true leaves, and large-leaf stage about 10 true leaves) of purple-leaf Chinese cabbage. *BraA02G001830*, *BraA03G004325* and *BraA07G003463* were almost not expressed in all plants.

**Figure S15 The phenotypes of wild-type *Arabidopsis thaliana* (Col-0) and *A. thaliana* 35S:*BraMYB114-GFP* overexpression line.**

**Figure S16 The interaction between *BraMYB114* and *BraTT8* in the nucleus of *Nicotiana benthamiana* epidermal cells.** Negative control: cYFP together with BraTT8-nYFP, BraMYB114-cYFP together with nYFP. Positive transformants of BraMYB114-cYFP together with BraTT8:nYFP.

**Figure S17 Yeast one-hybrid assays further verified the binding of BraMYB114 to the promoters of *BraANS.A01* and *BraDFR.A09*.** Positive control: pGADT7-p53 together with p53-His2. Negative control: pGADT7-BraMYB114 together with p53-His2. Positive transformants of pGADT7-BraMYB114 together with pHis2-BraANS.A01 (A) or pHis2-BraDFR.A09 (B) were further verified by spotting serial dilutions of yeast cells (1, 1/10, and 1/100) on TDO medium supplemented with 80 mM or 70 mM 3-AT, respectively. DDO: SD/-Leu/-Trp; TDO: SD/-His/-Leu/-Trp.

**Figure S18 Development of *BraMYB114* specific dominant markers.** *BraMYB114*-TY1 to *BraMYB114*-TY4 are the marker names of *BraMYB114*.

**Figure S19 Amplification of *BraMYB114*-specific molecular marker TY-3. (A)** ZBC-1 × W01-26 F_2_ segregating population. Both purple-leaf (‘ZBC-1’ type plants in F_2_ segregating population) and light purple-leaf plants (F_1_ type plants in F_2_ segregating population) contain the *BraMYB114* gene, which allows for the detection of a specific band. In contrast, green-leaf plants lack *BraMYB114*, resulting in no band being detected. **(B)** A total of 19 *B. rapa* lineswere examined, and none contained the *BraMYB114* gene; accordingly, no bands were detected.
